# Dysplastic *Ichthyosis Uteri*-like changes of the entire endometrium associated with a squamous cell carcinoma of the uterine cervix

**DOI:** 10.1186/1746-1596-1-8

**Published:** 2006-05-19

**Authors:** Oluwole Fadare

**Affiliations:** 1 Department of Pathology, Yale University School of Medicine, New Haven, CT, USA

## Abstract

Ichthyosis uteri is an exceedingly rare condition in which the entire surface of the endometrium is replaced by stratified squamous epithelium. Originally described as an endometrial response to iatrogenically-introduced caustic substances, similar changes have since been described in association with a variety of inflammatory conditions of the endometrium. We describe herein a heretofore undescribed example of a moderately differentiated squamous cell carcinoma of the uterine cervix associated with extensive ichthyosis uteri-like changes of the entire adjacent endometrium. Additionally, the squamous epithelium of the latter also showed multifocal changes diagnostic of a low-grade squamous intraepithelial lesion. The potential genesis of this composite of findings is discussed, as is the neoplastic potential of ichthyosis uteri. It is concluded that a squamous cell carcinoma of the cervix extended proximally into the endometrium, and that there was a colonization of a pre-existing ichthyosis uteri by associated human papillomavirus. The possibility of significant cervical pathology should be considered when plaques of squamous epithelium with low grade dysplastic changes are identified in an endometrial biopsy or curettage.

## Clinical history

A 38-year-old nulligravid female presented with complaints of a vague pelvic heaviness and a vaginal discharge. Her past medical history is significant for acquired immune deficiency syndrome (diagnosed 9 months previously) and Hepatitis A, B and C infections. Physical examination revealed a massively enlarged, barrel-shaped cervix with areas of ulceration on the ectocervix and a fungating mass protruding from the endocervical region. Following a biopsy, the patient underwent a type III radical hysterectomy, bilateral salpingo-oophorectomy and pelvic/paraaortic lymph node sampling. The procedure was well-tolerated and without any complications. She has had no evidence of tumor recurrence at last follow-up, 9 months after her surgery.

## Pathologic findings

### Macroscopic

**Figure 1:**
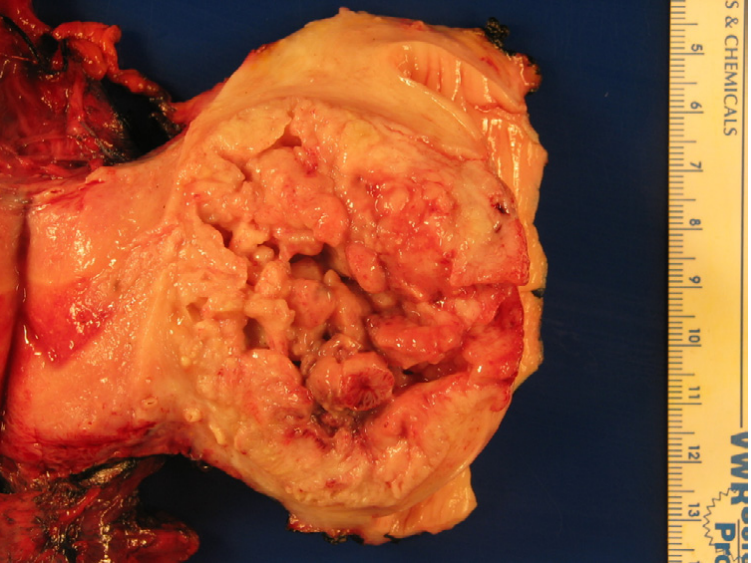
Gross appearance of the tumor showing a large exophytic mass obliterating the endocervical canal. The uterine fundus is on the left portion of the field.

Upon external inspection of the uterus, both the cervix and the inferior portion of the lower uterine segment were significantly enlarged. Sectioning revealed a 7 cm × 4 cm exophytic, friable, tan to tan-brown mass obliterating the endocervix and extending into the lower uterine segment (Figure 1). Sectioning revealed stromal invasion of the tumor to almost 100% of the cervical wall thickness. In the portions of the endometrium immediately proximal to the main mass, small soft satellite tumor nodules were present. The rest of the endometrium was tan-pink, flat and glistening but was otherwise unremarkable.

### Microscopic

**Figure 2:**
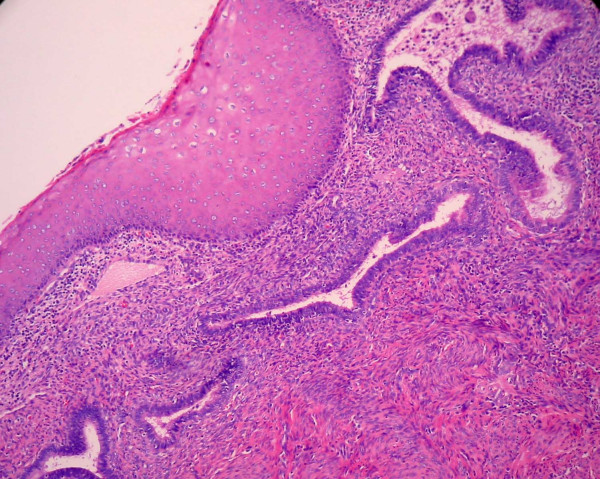
Areas consistent with ichthyosis uteri in which bland squamous epithelium overlies endometrial glands.

**Figure 3:**
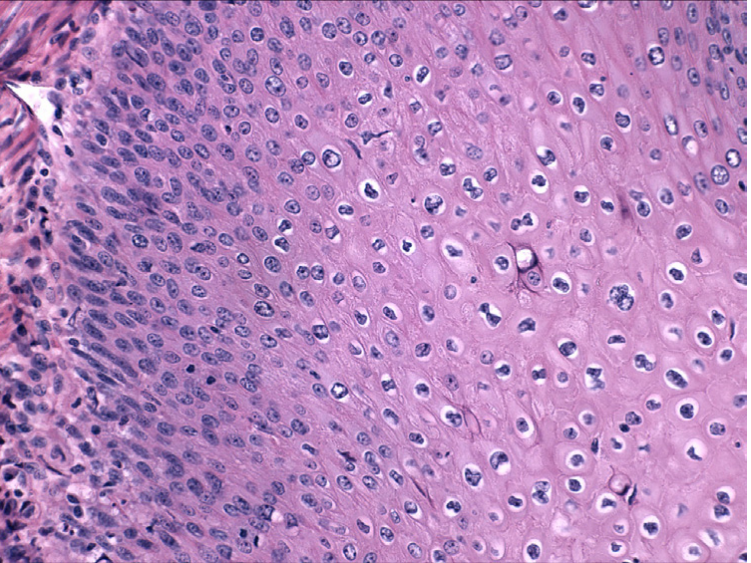
Areas of dysplastic epithelium overlying endometrial stroma.

**Figure 4:**
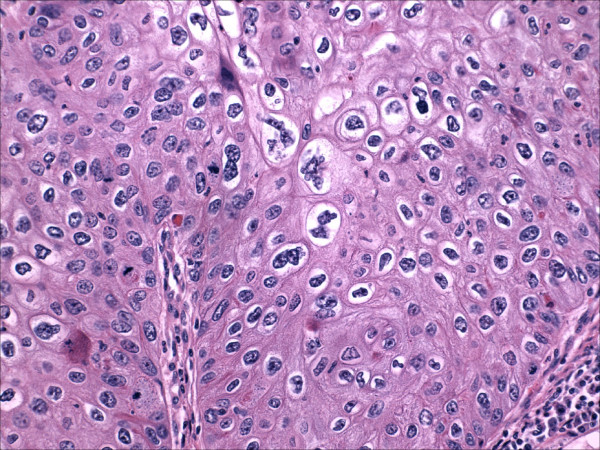
High power view of dysplastic squamous epithelium

The tumor was a moderately differentiated, large cell, keratinizing squamous cell carcinoma as previously described [1]. Notably, the epithelium retained prominent koilocytic changes throughout the tumor. The tumor was deeply invasive, extending to almost 100% of the cervical wall thickness. Perineural invasion was present. There was no evidence of extrauterine disease and the patient was assigned an International Federation of Gynecology and Obstetrics stage of 1B2. The endometrium was of the proliferative-type and there was no evidence of acute or chronic endometritis. Overlying the endometrium, however, was flat layer of mature, keratinizing squamous epithelium (Figure 2). The epithelium lacked a granular layer and was parakeratotic in most of the sections examined. In approximately half of the tissue sections, the cells displayed koilocytosis, nuclear hyperchromasia, nuclear enlargement, nuclear membrane irregularities and a moderate increase in nuclear-cytoplasmic ratio, i.e changes diagnostic of a low-grade squamous intraepithelial lesion in the lower genital tract (Figures 3 and 4). The sections showing the dysplastic changes were not exclusively from the inferior half of the uterine corpus. Immunohistochemical stains for human papillomavirus (HPV) was positive in both the cervical tumor and in the dysplastic squamous epithelium of the endometrium.

## Discussion

The term "ichthyosis uteri" was initially coined in 1885 by Zeller to refer to extensive squamous metaplasia of the surface endometrium following iatrogenically-introduced caustic substances such as formalin or iodine [2]. Since that initial report, the term "ichthyosis uteri" and the phenomenon it describes have become well accepted but has been used only sporadically in the literature [3-11]]. The case reported herein is a cervical squamous cell carcinoma associated with extensive ichthyosis uteri-like changes of the endometrium that, additionally, had superimposed low-grade dysplastic changes. This composite of findings may be explained in two, somewhat mutually exclusive ways: The first and most plausible explanation, and which formed the basis of the clinical diagnosis actually rendered, is that a squamous cell carcinoma originated in the cervix and the associated HPV extended proximally, colonizing a pre-existing ichthyosis uteri. Due to the distinct rarity of this composite of findings, it is hypothesized that the immunocompromised state of the patient contributed to this process. The second potential explanation is that within a background of extensive ichthyosis uteri, a squamous cell carcinoma developed in the lower uterine segment. Factors in favor of the former interpretation are 1) The central nidus of the mass was in the cervix, 2) The areas showing the highest degrees of stromal invasion were in the cervix 2) Primary squamous cell carcinomas of the endometrium are exceedingly rare and generally do not show koilocytic changes 3) At the patient's age of 38 years old, a cervical cancer is significantly more likely than an endometrial cancer. Although an argument can be made for a purely lepidic, surface extension of a cervical squamous cell carcinoma (and no pre-existing ichthyosis uteri), the absence of any high grade dysplastic or koilocytic changes in many segments of the endometrial squamous epithelium argues against this possibility. The case in the literature that most closely resembles our case was reported by Patton and Squires in 1962 [9]. The patient described in that report underwent a hysterectomy for severe pyometria. Microscopic evaluation showed extensive high-grade dysplasia of the cervix (with a focal area of microinvasion) and ichthyosis uteri of the endometrium. However, areas of the squamous epithelium in the endometrium also showed some degree of "cellular anaplasia", which the authors interpreted as a direct extension from the cervix. Pins et al described a somewhat similar case in which high-grade dysplasia of the cervix (and without invasion) extended proximally and coated the entire endometrium [12]. Both of these cases differ from the current case in that the dysplasia of the endometrial squamous epithelium in the latter was multifocal and low-grade, which argues against the possibility of a direct lepidic spread of her cervical tumor.

The second, albeit unlikely, potential genesis of this tumor (i.e a squamous cell carcinoma arose form ichthyosis uteri in the lower uterine segment) caused us to re-evaluate the literature on the neoplastic potential of ichthyosis uteri. In the experience of some authors [11], ichthyosis uteri lacks any malignant potential, and we encountered no evidence that directly suggests otherwise. However, the rarity of ichthyosis uteri hinders the interpretation of the significance of anecdotal cases of neoplasms arising from this entity. Presumably, longstanding, mature, plaque-like and even keratinizing squamous epithelium in the endometrium would be subject to the same potential changes as squamous epithelium in other visceral sites. A case of a benign papilloma arising in a background of extensive ichthyosis uteri was reported by Kucukali et al [4]. Similarly, malignancies have rarely been associated with ichthyosis uteri. Squamous cell carcinomas of the endometrium have been observed to arise either directly from [6] or in association with ichthyosis uteri [10]. Bewtra et al [3] recently described a case of endometrial adenocarcinoma covered almost entirely by a plaque-like, keratinizing mature squamous epithelium. The latter is in contrast to the plaque-like squamous differentiation which is rarely seen in endometrioid adenocarcinomas of the endometrium [13]; these changes are generally focal or multifocal and are not diffuse as is seen in ichthyosis uteri. Finally, ichthyosis uteri in the clinical course of case of endometrial adenocarcinoma was reported by Sikorowa in 1969 [8]. Although the number of cases of ichthyosis uteri associated with malignancies is disproportionately high relative to the overall number of cases reported, this is likely a bias created by the reporting of individual case reports. In our opinion, there is insufficient evidence to suggest that uncomplicated (i.e non-dysplastic) ichthyosis uteri has any intrinsic neoplastic potential.

In summary, a case of squamous cell carcinoma of the cervix with probable proximal extension into, and colonization of, a pre-existing ichthyosis uteri by associated HPV is described. The possibility of significant cervical pathology should be considered when plaques of squamous epithelium with low grade dysplastic changes are identified in an endometrial biopsy or curettage.

## Competing interests

The author(s) declare that they have no competing interests.

## Authors' contributions

Not Applicable
